# Should We Offer Universal Germline Genetic Testing to All Patients with Pancreatic Cancer? A Multicenter Study

**DOI:** 10.3390/cancers16223779

**Published:** 2024-11-09

**Authors:** Joan Llach, Irina Luzko, Julie Earl, Emma Barreto, Mercedes Rodríguez-Garrote, Marc Lleixà, Cristina Herrera-Pariente, Guerau Fernández, Jenifer Munoz, Laia Bonjoch, Tamara Saurí, Fabio Ausania, Teresa Ocaña, Lorena Moreno, Elia Grau, Josep Oriola, Maria Isabel Alvarez-Mora, Marta Herreros-Villanueva, Sergi Castellví-Bel, Francesc Balaguer, Luis Bujanda, Leticia Moreira

**Affiliations:** 1 Department of Gastroenterology, Hospital Clínic Barcelona, 08036 Barcelona, Spain; jllachr@clinic.cat (J.L.); luzko@clinic.cat (I.L.); lleixa@clinic.cat (M.L.); jmunozs@recerca.clinic.cat (J.M.); mocana@clinic.cat (T.O.); lomoreno@clinic.cat (L.M.); eggarces@iconcologia.net (E.G.); fprunes@clinic.cat (F.B.); 2 Centro de Investigación Biomédica en Red en Enfermedades Hepáticas y Digestivas (CIBEREHD), 08036 Barcelona, Spain; cristina.herrera@ciberehd.org (C.H.-P.); bonjoch@recerca.clinic.cat (L.B.); sbel@recerca.clinic.cat (S.C.-B.); 3 Institut d’Investigacions Biomèdiques August Pi i Sunyer (IDIBAPS), 08036 Barcelona, Spain; sauri@clinic.cat (T.S.); ausania@clinic.cat (F.A.); joriola@clinic.cat (J.O.); mialvarez@clinic.cat (M.I.A.-M.); 4 Campus Clínic, University of Barcelona, 08036 Barcelona, Spain; 5 Ramón y Cajal Health Research Institute (IRYCIS), The Biomedical Research Network in Cancer (CIBERONC), 28029 Madrid, Spain; julie.earl@live.co.uk (J.E.); emmabarretomelian@hotmail.com (E.B.); mercedes3110@yahoo.es (M.R.-G.); 6 School of Medicine and Health Sciences, University of Alcalá, Alcalá de Henares, 28805 Alcalá de Henares, Spain; 7 Department of Genetic and Molecular Medicine-IPER, Hospital Sant Joan de Déu, Institut de Recerca Sant Joan de Déu, Center for Biomedical Research Network on Rare Diseases (CIBERER), 08036 Barcelona, Spain; gfernandezi@sjdhospitalbarcelona.org; 8 Medical Oncology Department, Hospital Clinic and Translational Genomics and Targeted Therapies in Solid Tumors, IDIBAPS, 08036 Barcelona, Spain; 9 Department of General and Digestive Surgery, Hospital Clínic Barcelona, 08036 Barcelona, Spain; 10 Biochemistry and Molecular Genetics Department, CDB, Hospital Clínic de Barcelona, 08036 Barcelona, Spain; 11 Facultad de Ciencias de la Salud, Universidad Isabel I, 09003 Burgos, Spain; mhv@hgy.es; 12 Department of Gastroenterology, Hospital Donostia, 20014 San Sebastián, Spain; 13 Instituto Biodonostia, 20014 San Sebastián, Spain; 14 Department of Gastroenterology, Biogipuzkoa Health Research Institute, Centro de Investigación Biomédica en Red de Enfermedades Hepáticas y Digestivas (CIBERehd), Universidad del País Vasco (UPV/EHU), 20014 San Sebastián, Spain; luis.bujandafernandezdepierola@osakidetza.eus

**Keywords:** pancreatic cancer, genetic testing, germline pathogenic variant

## Abstract

Background: Pancreatic ductal adenocarcinoma (PDAC) is associated with a significant percentage of germline pathogenic variants (GPVs). Unlike in the United States, routine universal genetic testing is not performed in Europe. The aim of the study is to evaluate the diagnostic yield of germline genetic testing in all patients with PDAC. Methods: Individuals with newly diagnosed PDAC from three Spanish hospitals were enrolled, regardless of family history. Thirteen known susceptibility genes for PDAC were studied using a multigene panel or whole-exome sequencing. Results: One hundred seventy-nine PDAC patients underwent genetic testing. Fourteen (7.8%) had a GPV or likely pathogenic variant In the genes studied: six in *ATM*, six in *BRCA2*, one in *PALB2*, and one in *TP53*. Of these, seven (50%) did not meet the clinical criteria for genetic study and would have been classified as sporadic PDAC. Presenting with a personal history of any other neoplasm was associated with some GPV, with an odds ratio (OR) of 3.5 (1.1–11.5). A family history of PDAC and breast cancer was also associated with some GPV, with oRs of 3.7 (1.08–13.6) and 8.5 (2.6–26.6), respectively. None of the patients over 60 years without a relevant family history of malignancies presented a GPV associated with PDAC. Conclusions: In our PDAC cohort, a noteworthy number of GPVs were identified, and half of these patients would have been classified as sporadic based solely on clinical criteria. Genetic testing should always be considered, particularly in patients under 60 years or those with a history of other malignancies, especially where economic resources need optimization.

## Simple Summary

If we relied solely on clinical criteria for genetic testing, about half of pancreatic cancer patients with a germline pathogenic variant would be missed and classified as sporadic cancers. Patients over 60 years old with no relevant family history of cancer exhibited low probabilities of harboring any pathogenic variants. The age “<60 years” could serve as a cut-off point in regions where germline genetic testing is not routinely performed.

## 1. Introduction

Globally, the number of deaths, incident cases, and disability-adjusted life-years caused by pancreatic ductal adenocarcinoma (PDAC) has more than doubled from 1990 to 2017, and the increase in its incidence is likely to continue as the population ages [1]. PDAC is the seventh leading cause of cancer death in both sexes worldwide, and it is estimated that, by 2030, it will be the second cause of death from cancer in the United States of America [2,3]. 

Although most are associated with environmental factors, 10% of PDACs have a hereditary origin or some kind of family aggregation [4]. There are two clinical situations where familial predisposition to PDAC has been described: familial PDAC, in which a familial aggregation is observed without an identified hereditary cause, and hereditary PDAC, in which there is an association with a germline pathogenic variant (GPV) that carries an increased risk of developing this neoplasm. The extent to which deleterious GPVs contribute to PDAC risk in individuals without a family history of PDAC or other neoplasms is not well defined, but being able to identify all patients with a hereditary syndrome should always be a strategy to consider, since it can be very useful for both PDAC treatment and prevention [5,6]. 

The established PDAC susceptibility genes include *BRCA1*, *BRCA2*, *PALB2*, *ATM*, *CDKN2A/p16*, *PRSS1*, *STK11*, *APC*, *TP53*, *MLH1*, *MSH2*, *MSH6*, and *PMS2* [7,8]. The main promising targetable genetic alterations in PDAC are related to DNA damage-associated agents, and regarding treatment implication, the most well-established genes involved in this pathway are *BRCA1*, *BRCA2*, *PALB2*, and *ATM* [4]. Taking other studies into account, *BRCA2* shows the strongest association with PDAC among these genes, with a frequency of 1.9%, compared to a weaker association for *BRCA1* at 0.6% [9]. Some other studies have addressed the potential targeted therapy in these hereditary syndromes, suggesting a better response to platinum-based chemotherapy and to poly (adenosine diphosphate) ribose polymerase (PARP) inhibitors [10,11,12]. On the other hand, identifying families with these genetic alterations makes it easier to personalize prevention strategies based on risk (depending on each hereditary syndrome identified), and additionally, recent studies have demonstrated that PDAC surveillance in well-established groups may be an effective and survival-enhancing strategy for PDAC [13]. 

There is no consensus on the approach to germline genetic testing in newly diagnosed PDAC. The indication for germline testing is traditionally based on clinical criteria of the different associated syndromes, but recently, this approach has been changing for less restricted criteria, since GPVs have also been reported in ~5% of patients with apparently sporadic PDAC [9]. Recent National Comprehensive Cancer Network (NCCN) guidelines recommend offering gene testing for patients with newly diagnosed PDAC regardless of family history [14], and the American Society of Clinical Oncology guidelines also consider the role of genetic testing for patients with PDAC, even if family history is unremarkable [6]. However, this recommendation did not reach consensus in the International Cancer of the Pancreas Screening (CAPS) Consortium update [5]. In addition, the European Society for Digestive Oncology (ESDO) recommends germline testing only for patients who meet the clinical criteria for PDAC-associated hereditary syndromes [15]. 

The recent introduction of next-generation sequencing technology (NGS) has revolutionized the identification of GPV [16], allowing analysis costs to be simplified and reduced, as well as considerably increasing the information obtained. The use of multigene panels allows for the simultaneous sequencing of several genes potentially involved in the observed phenotype, although it also implies a challenge in the interpretation of the results by multiplying the number of genetic variants detected.

Up until now, in most European countries, a diagnosis of PDAC has not justified performing a germline genetic study unless a specific syndrome is clinically suspected [15]. Some studies have reported that the prevalence of GPV in the setting of PDAC is higher in young patients [17], although a cut-off point from which gene testing is considered cost-effective has not been established. 

The aim of the study is to evaluate the diagnostic yield of germline genetic testing in patients with newly diagnosed PDAC, independently of their personal and/or family history of other cancers. This study also aims to identify groups, specifically considering age, in which germline analysis should be considered. 

## 2. Materials and Methods

### 2.1. Study Design and Population

This is a retrospective study involving three tertiary Spanish hospitals: Hospital Clínic de Barcelona, Hospital Universitario de Donosti, and Hospital Universitario Ramón y Cajal. The study targets patients newly diagnosed with PDAC (incidental cases) who underwent germline genetic study regardless of family history or age, and the inclusion period spanned from 2014 to 2022. 

### 2.2. Data Recording

All patients were evaluated at high-risk gastrointestinal cancer clinics, where their personal and family cancer histories were thoroughly assessed.

Personal data: age, sex, alcohol consumption (at least 14 units/week), smoking habits (current, former, never), comorbidities, and oncologic history were recorded.

Family history of neoplasia (digestive and extra-digestive neoplasms, type of cancer, age, and degree of relationship) was recorded. Whenever possible, medical reports were requested to avoid recall bias.

The definition of classical criteria for germline testing was established as a patient who met the defined clinical criteria for PDAC-associated hereditary syndromes (including *BRCA1*, *BRCA2*, *PALB2*, *ATM*, *CDKN2A/p16*, *PRSS1*, *STK11*, *APC*, *TP53*, *MLH1*, *MSH2*, *MSH6* and *PMS2* genes).

### 2.3. Germline Genetic Analysis

A blood sample for biobanking was obtained from all patients newly diagnosed with PDAC either as part of the medical consultation at the high-risk clinic or at the time of the endoscopic ultrasound. Multigene panels or whole-exome sequencing were employed to analyze potential pathogenic variants (the technique used was based on the availability of each center; in 2 centers, a multigene panel was performed, while in the third center, exome sequencing was employed solely for research purposes). 

The panel study was conducted by amplifying the exons and flanking intronic regions of the genes using the Nextera Flex protocol for enrichment with Hereditary Cancer panel v2 probes for massively parallel sequencing on the MiSeq platform (Illumina, San Diego, CA, USA). This protocol demonstrates 100% sensitivity and specificity for single-nucleotide variants and a sensitivity of ≥80% and 100% specificity for insertions and deletions. The studied genes were: *BRCA1*, *BRCA2*, *ATM*, *PALB2*, *CDKN2A*, *STK11*, *APC*, *PRSS1*, *TP53*, *MLH1*, *MSH2*, *MSH6*, and *PMS2*. 

On the other hand, whole-exome sequencing was performed in cases where feasible, focusing on the study of the 13 mentioned genes. The variant detection protocol involved several key steps. Initially, read quality was assessed using FastQCv.0.11.5, followed by the removal of low-quality adapters and reads using cutadapt v1.13. The remaining reads were aligned to the human reference genome (hg19) with bwa-mem v.0.7.15, and low-quality mappings and duplicates were filtered out using BEDtools v.2.26.0 and Picard v2.9.0, respectively. Variant calling was performed using three softwares: DeepVariant v.0.10, GATK v.3.7, and Octopus v.0.6.3-beta. Only highly confident calls were annotated with public databases, namely: gnomAD r2.0.2, Clinvar, dbSNP_138, OMIM, and COSMIC, as well as with internal frequency databases. Clinvar, OMIM, and Cosmic were updated at the time of analysis. Mean sequencing coverage was >95× in all tested samples. Sequencing reads were considered of good quality only if they were >50 bp. For trimming purposes, a Phred Score of 20 or higher per base was used. Highly confident sequencing calls were selected, taking into account a total depth of coverage > 15.

We considered cases to have an uninformative genetic study when no relevant alterations were observed in the genes studied, including benign or likely benign variants and variants of uncertain significance (VUS). We considered a “pathological genetic study” in cases where pathogenic or likely pathogenic variants were identified in the genes studied. We considered a “pathological genetic study” in cases where rare (frequency < 1%), pathogenic or likely pathogenic variants were identified in the genes studied. Variant classification was carried out following American College of Medical Genetics and Genomics guidelines [18]. All VUS were discussed in expert committees involving geneticists and biologists to define them as accurately as possible. None of them were prioritized as candidate causative genetic variants.

### 2.4. Statistical Methods for Data Analysis

Statistical analysis was performed using the SPSS 23 version (SPSS Inc., Chicago, IL, USA, 2021). Quantitative variables were expressed as medians and interquartile ranges (IQRs) or means and SD depending on the distribution, and categorical variables were expressed as total number and frequencies (%). Comparisons between categorical data were performed with the chi-squared test or Fisher’s exact test; for continuous data, Student’s *t*-test for parametric and the Mann–Whitney U test for nonparametric data were used. A two-tailed *p* < 0.05 was considered statistically significant. Odds ratios (ORs) with 95% confidence intervals (CIs) were included to quantify the magnitude of the association. 

## 3. Results

### 3.1. Patient Selection and Clinical Characteristics

One hundred seventy-nine individuals with PDAC who underwent germline genetic testing were included. One hundred nine (60%) were men, and the median age at diagnosis was 58 years [interquartile range (IQR) 49–66]. Of the 179 individuals tested, only 19 (10.5%) met the clinical criteria for germline genetic study, while the remaining 160 did not meet the criteria for any hereditary syndrome associated with PDAC. The main characteristics are summarized in Table 1.

### 3.2. Outcomes of Germline Genetic Testing 

**Figure 1:**
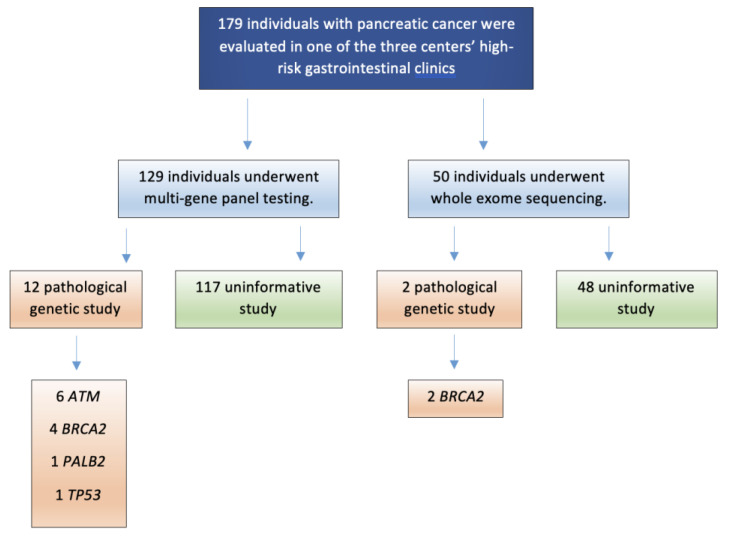
Flowchart of patients included in the study.

One hundred twenty-nine (72.1%) patients underwent multi-gene panel testing, while exome sequencing was performed in 50 (27.9%), according to the center that performed the study and the availability. A pathological genetic study (GPV or likely pathogenic variant) was identified in 14 individuals (7.8%, see Table 2), while 165 individuals had uninformative results. The genes most frequently associated with a GPV or likely pathogenic variant were *ATM* (6/179, 3.4%) and *BRCA2* (6/179, 3.4%), followed by *PALB2* (1/179, 0.6%) and *TP53* (1/179, 0.6%). No pathogenic variants were observed in *BRCA1*, *STK11*, *CDKN2A*, *APC*, *PRSS1,* or those genes associated with Lynch syndrome (see Figure 1).

### 3.3. Association Between Family Cancer History and the Presence of PDAC-Associated GPVs 

Most patients (13/14, 92.9%) with deleterious germline mutations had a family history of other cancers, but only seven (50%) GPV carriers had classical clinical criteria to rule out a hereditary syndrome. Eleven (78.6%) of the fourteen individuals with GPVs had a family history of breast cancer reported in a first- or second-degree relative, four (28.6%) had family history of PDAC, three (21.4%) of prostate cancer, and two (14.3%) of ovarian cancer. Table 3 shows the association between the presence of GPVs and the personal and family history of other malignances. Presenting with a personal history of any other cancer was statistically significantly associated with a GPV, with an OR of 3.5 (CI 1.1–11.6, *p* = 0.03). On the other hand, presenting with a family history of breast cancer and PDAC was statistically significantly associated with a GPV, with ORs of 8.5 (CI 2.6–26.6, *p* < 0.001) and 3.7 (CI 1.08–13.6, *p* = 0.044), respectively. Finally, and as expected, the presence of clinical criteria for Hereditary Breast and Ovarian Cancer Syndrome (HBOC) was associated with a pathological genetic study, with an OR of 32.8 (CI 7.1–150.9, *p* < 0.0001).

Of the seven patients without clinical criteria for genetic testing with a GPV identified, six (85.7%) presented with a GPV in *ATM*, and one (14.3%) in *BRCA2*.

### 3.4. Association of Age at PDAC Diagnosis with the Identification of GPVs

The median age at diagnosis for patients with PDAC who were identified as having GPV in a known PDAC susceptibility gene was 51 years (IQR 46–52), which was statistically significantly lower than the average age of patients without an identifiable susceptibility gene mutation (58 years, IQR 51–63; *p* = 0.02).

When we evaluated the performance of genetic study by age ranges, we observed a higher yield in early PDAC. Table 4 shows the frequency of GPVs observed according to age ranges. The younger the age, the higher the yield of the genetic study, with statistically significant differences observed in the “<35”, “<40”, and “<50” age groups. Furthermore, when we excluded the 19 patients with a clinical indication for genetic study based on clinical suspicion (Table 5), we also observed that younger age was associated with a higher diagnostic yield of GPV. We identified statistically significant differences in the “<55” and “<50” age groups without any suspected hereditary syndrome compared to older patients (*p* = 0.039 and *p* = 0.021, respectively). Additionally, it can be seen (in green) that the cutoff point from which the genetic study did not detect any new GPVs is from the “60-year-old group” onwards.

## 4. Discussion

In our study, 179 individuals with confirmed PDAC were included. Each patient underwent analysis of 13 genes known to increase the risk of PDAC, regardless of family history. Fourteen GPVs or likely pathogenic variants were identified, representing a prevalence of 7.8% (14 out of 179 individuals). Most of these variants were associated with genes linked to breast and ovarian predisposition syndromes. 

As expected, patients with a family history of other malignances, especially those with suspected HBOC, presented a high prevalence of GPVs or likely pathogenic variants compared to others (OR of 32.8). However, in the remaining patients with apparent sporadic PDAC, a non-negligible prevalence of GPVs was observed, reinforcing the need for consideration of genetic testing in all patients newly diagnosed with PDAC. 

Presenting a personal or family history (first- or second-degree) of breast cancer was associated with having a pathological genetic study, regardless of whether the clinical criteria for HBOC were met. Moreover, a family history of PDAC also was statistically significantly associated with the presentation of a GPV or likely pathogenic variant, unlike what has been described in previous studies. Shindo et al. [19] reported in their cohort that only 9% of individuals with GPVs had a family history of PDAC, and in the study of Bannon et al. [17], a family history of PDAC also was not associated with presenting a GPV. 

We observed a significant yield of genetic testing among patients with PDAC who did not present any suspected hereditary syndrome, with a prevalence of pathological genetic study of 4.4% (7 out of 160 individuals without clinical suspicion of hereditary syndromes). These patients with apparently sporadic PDAC often do not have an extensive family history of other cancers that would trigger consideration for germline gene testing. In this case, seven out of fourteen (50%) patients would not have been diagnosed based solely on their previous personal family oncologic history. 

Excluding patients with clinical suspicion of hereditary syndrome (in whom there is no doubt that the yield of genetic study is high), and according to our results, we believe that any patient with a recent diagnosis of PDAC should be considered for genetic study, and above all, those under 60. In our cohort, among patients over 60 years old, only two GPVs in *BRCA2* were identified, and in both cases, they exhibited clinical criteria indicative of suspicion for HBOC. This indicates that no patient over 60 years without clinical indications benefited from the study in our cohort. Bannon et al. [17] published a study in 2019 with over 800 patients with PDAC who underwent genetic testing, and they observed a higher number of GPVs in patients younger than 60 years (prevalence of 19%) compared with older individuals. Other studies also suggest that an early onset of PDAC may be an important risk factor for the presence of GPVs [20]. Thus, we consider that in areas where economic resources need to be optimized, this age cutoff point could be appropriate. 

Shindo et al. [19] published in 2017 a cohort of 854 patients with apparent sporadic PDAC in which they identified 33 (3.9%) GPVs (12 in *BRCA2*, 10 *ATM*, 3 *BRCA1*, 2 *PALB2*, 2 *MLH1*, 1 *CDKN2A*, and 1 *TP53*). The prevalence of GPVs observed in our cohort is slightly higher, and this could be because the median age of our cohort is lower than that of the other study (58 vs. 65 years). However, in both studies (in which the same genes were sequenced), the distribution of GPVs was similar, with a high percentage identified in genes associated with inherited breast and ovarian cancer. Our study presents results like those reported in the previous literature, in which a small but clinically important portion of PDAC was associated with pathogenic or likely pathogenic variants in known predisposition genes, regardless of family history. The GPVs detected in our cohort have clinical implications for the probands and their relatives. Thus, they may benefit from primary prevention strategies and represent a high-risk subgroup of patients to consider for surveillance strategies and for experimental targeted therapies [21]. Recent evidence supports the potential efficacy of PARP inhibitors or platinum agents in PDAC in stabilizing disease progression in individuals with *BRCA* germline mutations, and among these agents, olaparib is the most extensively researched and recognized [22].

This study has several strengths. This is a multicenter cohort involving three Spanish tertiary hospitals. To the best of our knowledge, this is the first study to evaluate the performance of germline genetic testing in a European cohort with PDAC. Similar studies had been published in North American centers [17,19,23,24], yielding comparable results, leading to updates in the latest American guidelines. However, our study showed that the performance of genetic testing is also very high in our setting, regardless of family history, and we suggest an age cutoff (<60 years) that could particularly benefit this, especially in resource-limited countries. Moreover, all patients were evaluated at high-risk gastrointestinal cancer clinics, obtaining a thorough personal and family history of cancer, and all germline variants were discussed in multidisciplinary committees to classify them correctly. It is important to note that a blood test for constitutional purposes always requires an oncogenetic consultation. However, in standard medical care, the availability of consultation slots is not guaranteed if all PDAC patients need to be tested.

On the other hand, there are some limitations. All patients were evaluated at a High-Risk Cancer Clinic, and although we attempted to ensure that patients with newly diagnosed PDAC were referred regardless of family history or age, there may have been a selection bias in this regard. In our cohort, there is a high percentage of young patients (median age 58 years), likely because such patients are more commonly referred to high-risk clinics compared to older individuals. 

## 5. Conclusions

In summary, in our PDAC cohort, a noteworthy number of GPVs were identified, and half of these patients would have been classified as sporadic based solely on clinical criteria. Genetic testing should always be considered and could be particularly useful in patients under 60 or those with a personal or family history of other malignances, especially in settings where economic resources need to be optimized.

## Figures and Tables

**Table 1:** Baseline characteristics of the study population (*n* = 179).

Male, *n* (%)	109 (60%)
Age, median (IQR)	58 years (49–66)
Age group, *n* (%)	
<60 years	123 (69%)
≥60	56 (31%)
Alcohol consumption, *n* (%)	
≥14 units/week	54 (30%)
No	109 (61%)
In Past or Unknown	16 (9%)
Smoking, *n* (%)	
Never	57 (31.8%)
Former	2 (1.12%)
Actual	120 (67%)
Personal history of other malignancies, *n* (%)	26 (14.5%)
Breast	5 (2.8%)
Kidney	5 (2.8%)
Melanoma	2 (1.1%)
Colorectal	2 (1.1%)
Bile duct	2 (1.1%)
Lymphoma	2 (1.1%)
Larynx	2 (1.1%)
Central Nervous System	2 (1.1%)
Prostate	2 (1.1%)
Cervix	1 (0.6%)
Lung	1 (0.6%)
Family history of malignancies (first- or second-degree), *n* (%)	89 (49.7%)
Breast	27 (15.1%)
Pancreatic	19 (10.6%)
Gastric	18 (10.1%)
Colorectal	19 (10.6%)
Prostate	11 (6.1%)
Hematological	10 (5.6%)
Ovarian	10 (5.6%)
Melanoma	4 (2.2%)
Kidney	4 (2.8%)
Indication for germline testing (clinical criteria of a hereditary syndrome), *n* (%)	19 (10.5%)
Presence of GPV, *n* (%)	14 (7.8%)

*n*, number; GPV, germline pathogenic variants; IQR, interquartile range.

**Table 2:** Deleterious GPV found in pancreatic cancer susceptibility genes (*n* = 14).

Case	Age, Years	Sex	Gene *	Nucleotide Change	Effect	Zygosity	FH of PDAC (First- or Second-Degree)	FH of Other Malignances	Germline Testing Clinical Indication **
1	33	M	*ATM*	c.6711_6715delGGAAA	Frameshift	Heterozygous	Yes	Lung (cousin), breast in elderly (≥50, maternal grandmother)	No
2	33	M	*PALB2*	c.3483delT	Frameshift	Heterozygous	No	Early (<50) breast (sister), lungs (paternal aunt)	Yes
3	34	F	*TP53*	c.733G>A	Missense	Heterozygous	No	Melanoma (sister, aunt), early breast (sister)	Yes
4	46	F	*BRCA2*	c.2701delT	Frameshift	Heterozygous	Yes	Breast in elderly (maternal aunt)	No
5	47	M	*BRCA2*	c.5116_5119delAATA	Frameshift	Heterozygous	No	Breast in elderly (mother)	No
6	48	M	*BRCA2*	c.3264dupT	Frameshift	Heterozygous	No	Early breast (daughter), prostate (father), breast and gastric (paternal cousins), gastric (maternal aunt), ovarian (great grandmother)	Yes
7	48	M	*ATM*	c.2098C>T	Nonsense	Heterozygous	Yes	None	No
8	54	M	*ATM*	c.8075T>A	Nonsense	Heterozygous	No	Colon in elderly (father), breast in elderly (paternal aunt).	No
9	56	M	*ATM*	c.8977C>T	Nonsense	Heterozygous	No	Leukemia (father), lymphoma (paternal uncle)	No
10	57	F	*BRCA2*	c.4243G>T	Nonsense	Heterozygous	No	Prostate (father and grandfather), bladder and early breast (mother), primary peritoneal carcinoma (brother), kidney (brother), colonic (aunt), bladder (cousin)	Yes
11	58	F	*ATM*	c.3576G>A	Splice site variant	Heterozygous	No	Bladder (father), tongue (uncle and grandmother), liver (paternal grandfather).	No
12	59	F	*ATM*	c.3802del	Nonsense	Heterozygous	No	Hodgkin lymphoma (son), colon (daughter), lungs (father), ovarian and early breast (mother), colon (maternal grandmother, maternal cousin, and maternal uncle), gastric (maternal cousin), prostate (grandfather)	Yes
13	67	M	*BRCA2*	c.9026_9030delATCAT	Frameshift	Heterozygous	No	Early breast (mother, daughter)	Yes
14	74	M	*BRCA2*	c.262_263delCT	Frameshift	Heterozygous	Yes	Early breast (two sisters)	Yes

GPV: germline pathogenic variant; FH: family history; PDAC: pancreatic ductal adenocarcinoma; M: male; F: female. * ATM (NM_000051.3), BRCA1 (NM_007294.3), BRCA2 (NM_000059.3), PALB2 (NM_024675.3) and TP53 (NM_000546.5). ** The clinical criteria for genetic testing were defined according to the ESDO guidelines (clinical suspicion of Peutz–Jeghers syndrome, hereditary breast and ovarian cancer, familial melanoma, Lynch syndrome, or hereditary pancreatitis) [15].

**Table 3:** Association between personal and family history of malignancies and GPV detection in patients with PDAC (*n* = 179).

	OR	95% (CI)	*p* Value
Personal history of other malignances (any)	3.5	(1.1–11.6)	0.03
Family history of other malignancies (first- or second-degree)			
Breast	8.5	(2.6–26.6)	<0.001
Prostate	3.6	(0.87–15.8)	0.06
Pancreas	3.7	(1.08–13.6)	0.044
Ovarian	3.6	(0.69–19.2)	0.15
Colorectal	1.3	(0.28–6.4)	0.66
Melanoma	0.98	(0.16–6.6)	0.70
Gastric	0.62	(0.076–4.993)	0.65
Clinical criteria for HBOC	32.8	(7.1–150.9)	<0.0001

GPV, germline pathogenic variants; PDAC, pancreatic duct adenocarcinoma; OR, odds ratio; CI, confidence interval, HBOC: hereditary breast and ovarian cancer syndrome.

**Table 4:** Frequency of GPV identified according to age ranges in the study population (*n* = 179).

**GPV identified, *n* (%)—in each age group.**	**Age Range (Years)**		***p* Value**
	<75	≥75	
	14/165 (8.5%)	0/14 (0%)	0.6
	<70	≥70	
	13/154 (8.4%)	1/25 (4%)	0.7
	<65	≥65	
	12/140 (8.6%)	2/39 (5.1%)	0.7
	<60	≥60	
	12/123(9.8%)	2/56 (3.6%)	0.15
	<55	≥55	
	8/63 (12.7%)	6/116 (5.2%)	0.07
	<50	≥50	
	7/39 (17.9%)	7/140 (5%)	0.008
	<45	≥45	
	3/18 (16.7%)	11/161 (6.8%)	0.14
	<40	≥40	
	3/8 (37.5%)	11/171 (6.4%)	0.001
	<35	≥35	
	3/4 (75%)	11/175 (6.2%)	<0.001

*n*, number; GPV, germline pathogenic variant.

**Table 5:** Frequency of GPVs identified according to age range in patients without clinical indication for germline testing (*n* = 160).

**GPV identified, *n* (%)—in each age group.**	**Age Range (Years)**		***p* Value**
	<75	≥75	
	7/146 (4.8%)	0/14 (0%)	0.402
	<70	≥70	
	7/137 (5.1%)	0/23 (0%)	0.268
	<65	≥65	
	7/124 (5.6%)	0/36 (0%)	0.145
	<60	≥60	
	7/109 (6.4%)	0/51 (0%)	0.064
	<55	≥55	
	5/56 (8.8%)	2/104 (2%)	0.039
	<50	≥50	
	4/35 (11.4%)	3/125 (2.4%)	0.021
	<45	≥45	
	1/15 (6.6%)	6/145 (4.13%)	0.649
	<40	≥40	
	1/5 (20%)	6/155 (3.9%)	0.083
	<35	≥35	
	1/2 (50%)	6/158 (3.5%)	0.002

*n*, number; GPV, germline pathogenic variant.

## Data Availability

The data that support the findings of this study are available from the corresponding author upon reasonable request.
